# Characteristics and outcomes of family-practice patients with coronavirus disease 2019: a case series

**DOI:** 10.1186/s13256-021-02963-7

**Published:** 2021-07-20

**Authors:** Stefania Dzieciolowska, Oumeet Ravi, Roland Grad

**Affiliations:** 1 Faculty of Medicine, McGill University, Montreal, QC H3A 0G4 Canada; 2 Herzl Family Practice Centre, 3755 Cote Ste Catherine Road, Montreal, QC H3T 1E2 Canada

**Keywords:** Community medicine, Family medicine, General practice, Primary care, Epidemiology, SARS-CoV-2

## Abstract

**Background:**

The clinical history and outcomes of coronavirus disease 2019 among people not hospitalized is not yet well characterized. To better inform clinical evaluation, we set out to characterize the natural history of coronavirus disease 2019 in primary health care.

**Methods:**

Case series of all patients rostered to physicians at a university-affiliated Family Medicine clinic. Cases met the Centers for Disease Control and Prevention definition of coronavirus disease 2019 from March 1 to May 21 2020.

**Results:**

In total, 89 patients meeting Centers for Disease Control and Prevention criteria for coronavirus disease 2019 were documented. Their average age was 55.6 years (range 6–95 years), and all but one was symptomatic. Fifty-seven cases (64%) had a polymerase chain reaction test for coronavirus disease 2019, of whom 77.2% tested positive. Thirty cases (33.7%) reported contact with a confirmed or probable case of coronavirus disease 2019. Based on the Charlson Comorbidity Index, 28 cases (31.5%) had no comorbid conditions. The median number of days from symptom onset to first polymerase chain reaction test was 6 days (interquartile range 2.3–11 days). The median duration of fever was 3.5 days (interquartile range 1–7 days). Twenty-four cases (27%) visited the Emergency Department, and 10 were admitted to hospital. The median number of days between symptom onset and first Emergency Department visit was 8 days (interquartile range 3.5–27 days).

**Conclusions:**

At the start of this pandemic, the implementation of basic measures such as diagnostic testing was delayed. If we are to improve our control over the spread of coronavirus disease 2019, we will need to substantially reduce the time from symptom onset to diagnostic testing, and subsequent contact tracing. To minimize unnecessary Emergency Department visits, we propose a testable strategy for Family Medicine to engage with coronavirus disease 2019 patients in the acute phase of their illness.

**Supplementary Information:**

The online version contains supplementary material available at 10.1186/s13256-021-02963-7.

## Background

The novel coronavirus arose in late 2019, but soon became a worldwide threat as new cases emerged at rapid rates. The coronavirus, designated severe acute respiratory syndrome coronavirus 2 (SARS-CoV-2), was initially identified in Wuhan, China [1]. As the months progressed, the virus spread to almost all continents, resulting in the declaration of a pandemic by the World Health Organization (WHO) [2]. In Canada, the coronavirus disease 2019 (COVID-19) virus continues to pose a major threat. Generally, COVID-19 should be considered primarily in patients with new-onset fever with or without respiratory symptoms, and can often be accompanied by other symptoms. Public Health Canada categorized symptom severity based on symptoms that were most frequent, less frequent, and rare, and determined that the most prominent symptoms included fever, cough, shortness of breath, fatigue, loss of appetite, and loss of smell and/or taste [3]. While there are no specific clinical features that can reliably distinguish COVID-19 from other viral respiratory infections, the preferred diagnostic test for COVID-19 is done through nucleic acid amplification testing (NAAT) with polymerase chain reaction (PCR) from samples collected by nasopharyngeal swabs. In 2020, the Centers for Disease Control and Prevention (CDC) issued guidelines that defined COVID-19 patients as either probable or confirmed [4].

The first cases of COVID-19 in the Canadian province of Quebec were identified at the end of February 2020. On March 13 2020, a state of health emergency was declared. As of April 8 2020, Quebec adopted a case definition for COVID-19 [5]. In the first wave, during which this study was conducted, the earliest data showed that the overall number of daily deaths in Quebec was 1 on March 18 2020, compared with 61 deaths on May 21 2020. In Quebec, the city of Montreal suffered the highest rates of mortality [6].

As of late April 2021, the province of Quebec had over 344,000 confirmed COVID-19 cases and about 10,000 deaths [6]. Montreal had almost half of these cases, and was an early epicenter of COVID-19 in Canada. In Quebec, a COVID-19 Biobank was established but restricted data collection to severely ill patients in hospital [7]. However, many individuals with COVID-19 and non-severe illness presented for care to their family physician. To our knowledge, there is no case series on patients presenting to family physicians that describes the course of COVID-19 in the community setting. Hence, we have limited information on the natural history of these cases. We conducted this study to describe the clinical course of such cases to enable clinicians in primary health care to better evaluate and counsel their patients about COVID-19.

## Methods

### Study design

Case series of all patients rostered to physicians at the Herzl Family Practice Centre in Montreal (henceforth “the clinic”). Located in West-Central Montreal, this clinic is a McGill University-affiliated Family Medicine Group Practice with approximately 31,000 registered patients. The family medicine clinic is composed of a wide variety of providers at any given time: 55 physicians, 50 residents, 5 nurses, 1 nurse-practitioner, 2 kinesiologists, 2 nutritionists, 5 psychologists, 2 social workers, and a variable number of nursing and medical students.

### Aims/objectives

Our primary aim was to describe the natural history of COVID-19 in patients followed in a family-practice outpatient setting. Second, we wished to describe these cases with enough detail to allow others to make comparisons with their own practice.

### Inclusion and exclusion criteria

Our sampling frame was restricted to patients registered to any physician at the clinic who met the case definition of COVID-19 disease as proposed by the CDC [4]. Patients not registered at the clinic or who did not meet the CDC criteria for COVID-19 illness were excluded. The CDC definition of COVID-19 disease requires cases to fulfill either the clinical or the laboratory criteria [4] (Additional file 3):

- Clinical criteria (must have any one of the following):
  1. At least two of the following symptoms: fever (measured or subjective), chills, rigors, myalgia, headache, sore throat, or new olfactory and taste disorder(s).
  2. At least one of the following symptoms: cough, shortness of breath, or difficulty breathing.
  3. Severe respiratory illness with at least one of the following: clinical or radiographic evidence of pneumonia or acute respiratory distress syndrome (ARDS), and having no alternative more likely diagnosis.
- Laboratory criteria (must have any one of the following):
  1. Confirmatory laboratory evidence: detection of severe acute respiratory syndrome coronavirus 2 ribonucleic acid (SARS-CoV-2 RNA) in a clinical specimen using a molecular amplification detection test.
  2. Presumptive laboratory evidence: detection of specific antigen in a clinical specimen, or detection of specific antibody in serum, plasma, or whole blood indicative of a new or recent infection.

### Data collection

**Fig. 1:**
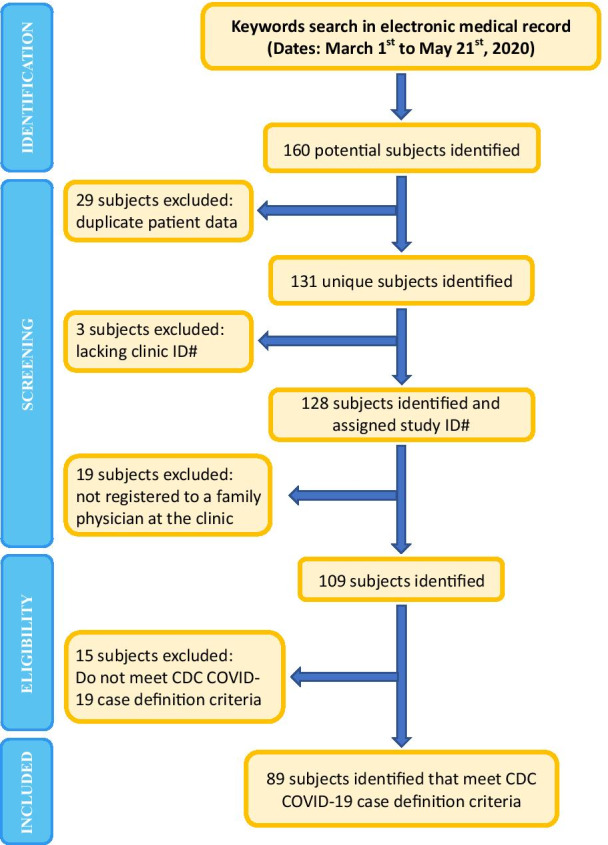
Case identification

We identified cases in a search of the clinics’ electronic medical record from March 1 to May 21 2020. Our search was done on May 21 2020, for keywords in the problem/diagnosis field. Relevant keywords were those related to coronavirus (Additional file 1). From this search, we generated our initial list of 160 subjects. Among this list, 29 duplicate entries were then excluded in addition to 3 patients with no clinic identifier number. This resulted in a list of 128 unique subjects. At this point, the names of patients were removed to maintain their confidentiality. Following this review, 19 subjects were removed from the study as they were not officially registered to a family physician at the clinic, resulting in a list of 109 remaining subjects. Lastly, 15 subjects were removed as they were only inquiring about the virus and were asymptomatic with no confirmed laboratory results, and met epidemiological criteria (contact 1–6), thus only partially fitting the CDC case definition. Finally, 89 subjects remained (Fig. 1). We then performed a chart review in which clinical information on each case was extracted and scrutinized.

With regard to COVID-19 testing practices and processing, in the first wave, testing centers for COVID-19 were designated by the provincial government and were largely based in hospitals/emergency departments (ED). When an outpatient wanted to have a SARS-CoV2 PCR swab done, the Montreal health hotline (InfoSante 811) directed the individual to the nearest test site in the community.

We did not seek to collect data on any specific symptoms. At the time of this study (first wave), family physicians had no specific list of symptoms to define this illness. Therefore, any and all symptoms on chart review were included in the data collection. These included anosmia, belching, chest discomfort, chest pain, chills, congestion, constipation, cough, decreased appetite, diarrhea, dizziness, dry cough, dyspnea, dysuria, fatigue, fever, headache, indigestion, joint pain, leg pain, lightheadedness, malaise, myalgia, nasal drip, nausea, neck pain, ocular symptoms, otalgia, palpitations, phlegm, rhinorrhea, shortness of breath, shortness of breath on exertion, somnolence, sore throat, sweats, tachycardia, tachypnea, tickling in throat, tinnitus, tremors, vomiting, and weakness.

No patients were interviewed in this study, but when they had reportedly visited the emergency department in the adjacent hospital with which the clinic is affiliated, we verified this information. We could not verify if patients presented to an ED other than the one affiliated to this clinic. The date of contact (by phone or in person) between patient and primary care provider was not noted. We chose to focus on the date of symptom onset as a primary time point, and then calculated the median number of days between symptom onset and first ED visit. We did not set out to determine if the ED visit occurred before or after the date of contact in the clinic. No data on socioeconomic status were collected.

Two investigators undertook data extraction independently. This involved a search for information on variables such as comorbid conditions as defined by the Charlson Comorbidity Index [8]. We sought inconsistencies in our extracted data during two review meetings. Disagreements were then resolved by discussion.

### Data analysis

We used descriptive statistics to characterize cases based on the following variables: sex, age, PCR testing, number of days between symptom onset and PCR testing, contact type, number of days between symptom onset and any ED visit, any hospitalization, medication prescribed for COVID-19 in the outpatient setting, and comorbidities using the Charlson Comorbidity Index [https://www.mdcalc.com/charlson-comorbidity-index-cci#evidence]. We reported event rates as proportions and described continuous variables using frequency counts and measures of central tendency. No analyses for statistical significance were performed given the descriptive nature of this study.

## Results

### Demographics

**Fig. 2:**
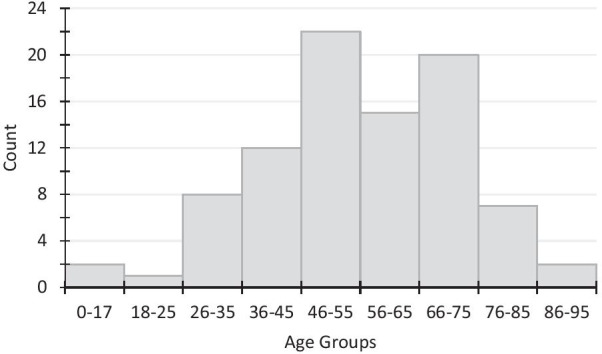
Age distribution

By sex, Table 1 describes the age, presence of symptoms, height, and weight of our cases. Symptoms were reported in all but one person. In Fig. 2, we further describe cases in a frequency distribution of their age.

**Table 1:** Demographics

	Total (89)	Male (39)	Female (50)
Number of cases	89	39	50
Symptoms (any)	88	39	49
Asymptomatic PCR+	1	0	1
Average age (years)	55.6	57.5	54.1
Average height (m)	1.61	1.69	1.53
Average weight (kg)	76.5	82.4	71.5

N.B.: Height data available for 33 cases

Weight data available for 68 cases

PCR: polymerase chain reaction, M: meters, Kg: kilograms

### PCR testing

**Fig. 3:**
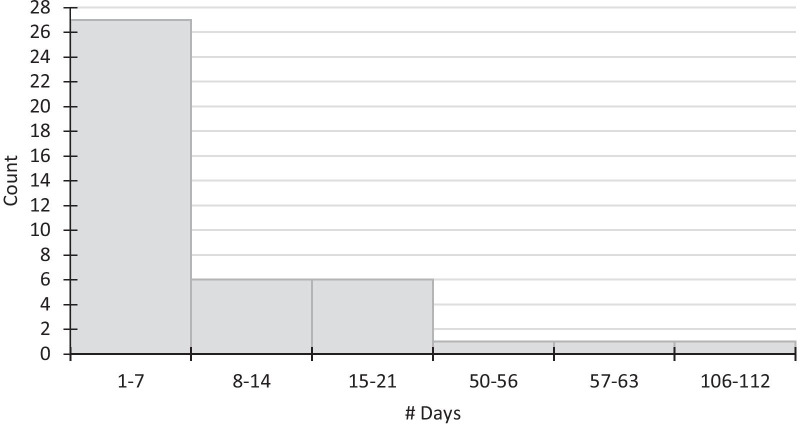
Distribution of days from symptom onset to PCR test

Of the 89 cases, 64% had a PCR test for COVID-19, of which 77.2% tested positive, 19.3% tested negative, and 3.5% had no PCR test result in the chart (Table 2). Among cases who were tested, we were interested in knowing the following: how soon cases went to get PCR testing following onset of symptoms, how many days it took for cases to become negative based on a follow-up PCR test, and how many days of fever cases experienced during their illness. Given that the time from symptom onset to PCR testing was a median of 6 days and this variable was not normally distributed, we further describe this distribution in 7-day bands in Fig. 3.

**Table 2:** Diagnostic testing and duration of fever

	Total (89)	Male (39)	Female (50)
PCR test result (*n*)	64.0% (57)	66.7% (26)	62.0% (31)
% Positive	77.2% (44)	84.6% (22)	71.0% (22)
% Negative	19.3% (11)	11.5% (3)	25.8% (8)
% Missing result	3.5% (2)	3.9% (1)	3.2% (1)
Symptom onset->PCR (days)			
MIN	1	–	–
25th percentile	2.25	–	–
Median	6	–	–
75th percentile	11	–	–
MAX	111	–	–
PCR POS->PCR NEG (days)			
MIN	12	–	–
25th percentile	18	–	–
Median	29	–	–
75th percentile	43	–	–
MAX	73	–	–
Fever (days)			
MIN	1	–	–
25th percentile	1	–	–
Median	3.5	–	–
75th percentile	7	–	–
MAX	22	–	–

N.B.: Symptom onset->PCR test: data available for 42 cases

POS->NEG test: data available for 12 cases

Fever: data available for 60 cases

PCR: polymerase chain reaction, MIN: minimum, MAX: maximum, POS: positive, NEG: negative

### Hospital-based services

Though our focus was outpatient family practice, we also wanted to know about the use of hospital-based health services. Of 89 cases, 24 (27%) visited the ED of the hospital to which the clinic is attached. The median number of days between symptom onset and ED visit was 8 days (Table 3). Ten (11%) of the 89 cases were hospitalized, where one case died (Table 3). Compared with cases who did not obtain diagnostic testing, cases who were PCR tested were also more likely to seek medical care in the ED.

**Table 3:** Emergency department visits and hospitalization

	Total (89)	Male (39)	Female (50)
Cases with ED visit % (*n*)	27.0% (24)	33.3% (13)	22.0% (11)
Cases with ED visit and PCR test % (*n*)	22.5% (20)	25.6% (10)	20.0% (10)
PCR+ % (*n*)	80% (16)	100% (10)	60% (6)
PCR− % (*n*)	15% (3)	0% (0)	30% (3)
PCR result missing % (*n*)	5% (1)	0% (0)	10% (1)
Symptom onset -> ED visit (days)			
25th percentile	3.5	–	–
Median	8	–	–
75th percentile	26.5	–	–
Hospitalized cases % (*n*)	11.2% (10)	15.4% (6)	8.0% (4)
Deaths	1	0	1

ED: emergency department, PCR: polymerase chain reaction, MIN: minimum, MAX: maximum, POS: positive, NEG: negative

### Patient contact type

**Fig. 4:**
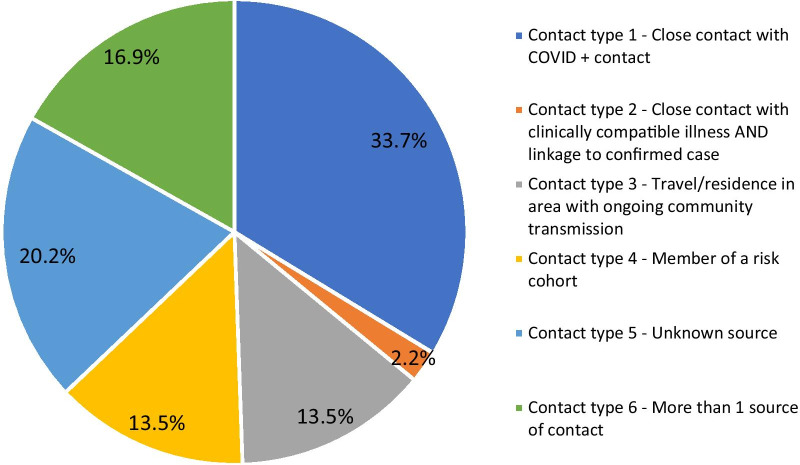
Case contact type

Given the importance of contact tracing, we examined the types of contact reported by cases with individuals in the community who may have infected them. In Fig. 4, we describe six forms of contact inspired by CDC epidemiologic criteria for COVID-19.

### Treatment

In 38 cases, medications were prescribed. These were antibiotics, hydroxychloroquine, and corticosteroids (oral, inhaled, or intravenous) (Table 4).

**Table 4:** Drug treatment

	Prescriptions (*N *= 38 unique patients)
Antibiotics	25*
Hydroxychloroquine	10
Steroids (oral/inhaled/intravenous)	9

*21 of 25 received azithromycin

### Comorbidity

**Fig. 5:**
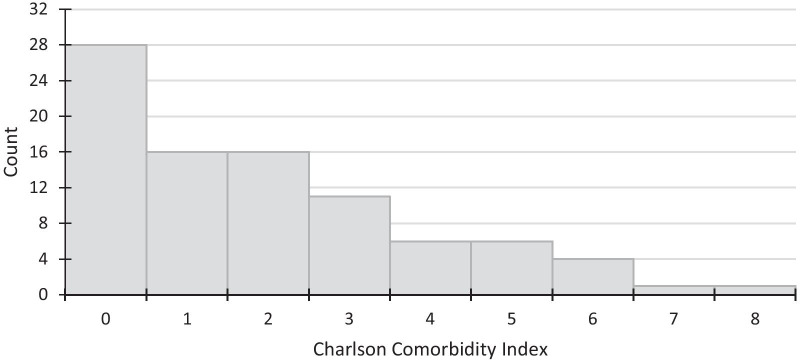
Distribution of Charlson score

Finally, given how preexisting health conditions can influence patient outcomes in the context of COVID-19, we characterized cases according to the Charlson Comorbidity Index and plotted the distribution of their Charlson score in Fig. 5. The median Charlson Comorbidity Index score was 2.

## Discussion

We conducted this case series to better understand the clinical course of COVID-19 in the community and, in so doing, help clinicians better counsel patients and families about what to expect regarding this disease. To our knowledge, as of August 19 2020, no case series existed on the natural course of COVID-19 in the community setting in the context of family practice. We confirmed this gap in the literature when, aided by an academic librarian, we systematically searched for case series and found no similar study. Other work has revealed associations between comorbid conditions and mortality [9], as well as predictors of intensive care unit (ICU) admission for COVID-19 [10].

Among 89 cases of COVID-19 who were known to a family medicine clinic, almost all were symptomatic. This finding is expected, as acutely infected but asymptomatic people do not typically seek medical attention. The majority of cases were in the age range of 46–85 years. While we know now that individuals aged over 75 years are more likely to become severely ill affected by the COVID-19 illness, it is possible that, due to the physical closure of many clinics and the transition to virtual care, individuals over 75 years who were less familiar with online care were also less likely to consult, compared with those who were younger.

Among the 89 cases, the average weight was 76.5 kg, based on 68 individuals, and the average height was 1.61 m, based on 33 individuals. From these data, we see that physicians record height less often than weight. As a higher body mass index (BMI) was thought to be an important risk factor for severe COVID-19 illness, this omission is clinically important [11–13]. In one early study, conducted from 1 March to 2 April 2020, among 4103 patients with COVID-19 at an academic health system in New York City, BMI  > 40 was the second-strongest independent predictor of hospitalization, after old age [11].

In our study, 64% of cases had a PCR test for COVID-19 and 77.2% tested positive. While one-quarter did not get tested, they were considered probable cases based on the CDC definition. The median number of days from symptom onset to obtaining a PCR test was 6 days; however, as the distribution was not uniform, we assessed the frequency distribution of this variable in 5-day bands (Fig. 3). While the majority of cases had presented for PCR testing within the first week of illness (*n *= 27), many waited until the second (*n *= 6) or third (*n *= 6) week of illness before getting tested. Furthermore, three cases tested positive more than 50 days since symptom onset, which was observed through incidental testing on presenting to the hospital for an issue unrelated to COVID-19. When it takes 6 days to get a COVID-19 test, patients are well past their peak infectiousness. In a study by Lavezzo *et al*., COVID-19 cases had an infectious period, as measured by viral load, of 3.6–6.5 days, with infectiousness peaking on the day of symptom onset [14]. In the first wave, an urgent need existed for innovative testing and contact tracing strategies such as saliva collection to enable earlier identification of cases [15].

While most individuals did not have a follow-up PCR test, 12 cases did have a follow-up negative PCR. Among these 12 cases, the median number of days to getting this second test was 29 days (Table 2). The observed delay in testing was in part due to the limited availability of tests in March and April 2020. We were interested in verifying the time between the first positive PCR and first negative PCR tests as it was observed that, during the first wave of the pandemic, some cases remained PCR positive for a long time, raising a concern for persistent infectivity. To this day, the occurrence of repeated positive tests for SARS-CoV-2 in recovered COVID-19 patients remains poorly understood. It has been suggested to be related to false-negative tests, false-positive tests, or reactivation or reinfection with COVID-19, but the mechanism leading to these persistently positive cases remains unclear [16].

The COVID-19 infection is often compared to influenza-like illness. In one review of the clinical course of influenza, fever was observed in 34.9% of individuals [17]. By comparison, in our study of COVID-19 cases, fever was observed in 67.4% of individuals. The higher rates of fever detected in our study may be due to a variety of factors such as the fact that we do not know the true infectivity rate of COVID-19 as well as due to the possibility that we did not have information on asymptomatic patients who were positive for COVID-19 and not tested. Whereas this aforementioned review included studies of healthy volunteers with objectively measured fever, our series focused on a chart review of patients in family practice whose fever was not objectively measured [17]. In another review, authors looked at the clinical spectrum and natural history of human influenza where fever was reported in 84.7% of confirmed cases of A(H1N1) [18]. In this review, fever was “reported to last approximately 5 days” [19]. In comparison, among our 60 cases who were symptomatic with fever, this symptom lasted for a median of 3.5 days (interquartile range 1–7 days).

About one-quarter of our cases visited the ED, which was objectively verified through scrutiny of medical records early in the pandemic, and 11.2% of cases were hospitalized. In a prospective cohort study conducted in the outpatient setting in Maryland between April 21 and July 23 2020, authors identified that 11.0% of participants presented to the ED, and 7.6% required hospitalization [20]. While the reported rates of ED visits and hospitalizations were lower in this study in comparison with ours, it is important to note that, at the time that our study was conducted, Montreal was an early epicenter of COVID-19 in Canada. Cases presented to the ED after a median of 8 days following symptom onset. Cases who underwent PCR testing were also more likely to visit the ED. This can be understood given that, at the time of this study, in-person office visits were not available for people with suspected or confirmed COVID-19.

### A response strategy in family medicine?

To minimize unnecessary ED visits for this disease, we encourage research on the effect of more intensive care in the primary care setting. Imagine if, early in the disease, a family physician referred their patient for testing to reduce the chance of community spread. Then, for confirmed or suspected cases, follow-up could be provided, for example, at days 3, 5, and 7 from symptom onset, and remote monitoring could be done as per the adult primary care COVID-19 assessment pathway [21]. The purpose of closer follow-up would be to assess for symptoms such as dyspnea at rest, as well as to obtain a measurement of oxygen saturation with a pulse oximeter that could be delivered to patients’ homes, as the predictive value of outpatient oxygen saturation values has been observed in patients with COVID-19 [22, 23]. For patients without hypoxia, reassurance to remain at home would be indicated. For those with an oxygen saturation of ≤ 94% on room air, an in-person evaluation or admission through the ED would then be warranted [24].

As contact tracing was important in the context of understanding the early spread of COVID-19 in the community setting, it was interesting to observe that the majority of patients had contact with a COVID-19-positive individual in addition to another source of contact. In fact, there was evidence of possible family spread of COVID-19; however, we only classified contact type as defined by the CDC as our intent was to better understand the type of exposure. In the prospective cohort study in Maryland, 40.2% of cases had contact with a confirmed COVID-19 patient, and 21.6% had contact with someone displaying symptoms concerning for COVID-19 [20]. This finding supports the need for earlier testing and stronger social distancing policies to more rapidly isolate COVID-19 cases, thus further decreasing spread of the virus. To meet rising demands in testing, healthcare authorities will require alternative methods such as self-sampling kits as studied in Washington, where self-collected samples in ambulatory clinics were 89.8% sensitive for tongue samples, 94.0% sensitive for nasal samples, and 96.2% sensitive for mid-turbinate samples [25].

As one-quarter of the patients in this study received an antibiotic prescription for their COVID-19 illness, it is important to question whether physicians were “choosing wisely.” At the time that this study was conducted, there was no formal policy to guide prescribing of antibiotics or steroids. throughout the pandemic, multiple studies emerged suggesting the use of azithromycin and Plaquenil as possible treatments for patients affected by COVID-19. Given the difficulty of examining patients and decreased access to chest x-ray in the early days of the pandemic, along with a fear of missing a secondary bacterial infection, the observed prescribing rate of antibiotics seems justifiable.

Finally, in our study, 28 cases (31.5%) had no comorbid conditions, and the median Charlson score was 2. This is similar to the prospective cohort study of outpatient cases in Maryland, where the median Charlson Comorbidity Index (IQR) was also 2 (1–3) [20].

### Limitations

One CDC study found that about ten times as many people have been exposed to the novel coronavirus than are reported as cases [26]. While we set out to describe the natural history of COVID-19 disease in patients followed in family practice, asymptomatic cases were not included as well as cases who sought urgent care at other sites and did not inform their family doctor. Consequently, we likely underestimated the extent of ED use and hospitalization. Although we observed one death, we do not know how many cases eventually succumbed to their illness, as charts were reviewed in a cross-sectional manner.

## Conclusion

At present, we are now just learning about the illness experiences of people with COVID-19 in community settings. If we are to improve our performance with respect to basic public health interventions such as contact tracing, the time from symptom onset to PCR testing will need to be substantially reduced. There is an urgent need in primary care for innovative strategies for diagnostic testing and therapeutic interventions proven to enhance outcomes for people with COVID-19.

## Supplementary Information

**Additional file 1.** Keywords used in the search for cases.

**Additional file 2.** STROBE checklist for cross-sectional studies.

**Additional file 3.** Center for Disease Control COVID-19 case definition.

## Data Availability

The datasets used and/or analyzed during the current study are available from the corresponding author on reasonable request. If other case series come to our attention, we will attempt to pool our results in a systematic review or meta-analysis of case series. Prior to undertaking such additional work, we will recontact our Research Ethics Board to discuss a submission of an amendment, and any need for a Data Transfer Agreement.

## Abbreviations

COVID-19: Coronavirus disease 2019
ED: Emergency Department
CDC: Centers for Disease Control
PCR: Polymerase chain reaction
MIN: Minimum
MAX: Maximum
POS: Positive
NEG: Negative
