# Supplementary material for: Characteristics and outcomes of family-practice patients with coronavirus disease 2019: a case series
Source: J Med Case Rep. 2021 Jul 20;15:393. doi: 10.1186/s13256-021-02963-7 (PMC8290383; doi:10.1186/s13256-021-02963-7)
Supplement: Supplementary file 1 — Additional file 1. Keywords used in the search for cases. [file 13256_2021_2963_MOESM1_ESM.docx]

**Additional File 1**

| coronavirus |
| --- |
| Coronavirus infection, unspecified site |
| COVID |
| COVID |
| COVID -19 |
| covid + |
| covid 19 |
| COVID 19 |
| COVID 19 infection |
| covid 19 outpatient |
| covid 19 positive |
| COVID 19 Situation |
| covid infection |
| COVID patient |
| covid pos |
| COVID positive |
| COVID SITUATION |
| COVID- 19 |
| COVID- 19 situation |
| COVID-19 |
| Covid-19 confinement |
| COVID-19 exposure |
| COVID-19 Infection |
| COVID-19 Positive (April 28, 2020) |
| COVID-19 positive (March 15, 2020) |
| Covid-19 situation |
| COVID+ |
| COVID19 |
| COVID19 |
| COVID19 CONFIRMED CASE |
| COVID19 INFECTION |
| COVID19 INFECTION, RECOVERING |
| COVID19 Pneumonia |
| COVID19 positive, residual cough |
| exposure to coronavirus |
| GAD exacerbated in the context of COVID-19 |
| Health concern - COVID-19 |
| Infection à covid 19 |
