# Supplementary material for: Characteristics and outcomes of family-practice patients with coronavirus disease 2019: a case series
Source: J Med Case Rep. 2021 Jul 20;15:393. doi: 10.1186/s13256-021-02963-7 (PMC8290383; doi:10.1186/s13256-021-02963-7)
Supplement: Supplementary file 3 — Additional file 3. Center for Disease Control COVID-19 case definition. [file 13256_2021_2963_MOESM3_ESM.docx]

# **Coronavirus Disease 2019 (COVID-19) - 2020 Interim Case Definition, Approved April 5, 2020**

1. **Clinical Criteria**

At least two of the following symptoms: fever (measured or subjective), chills, rigors, myalgia, headache, sore throat, new olfactory and taste disorder(s)

**OR**

At least one of the following symptoms: cough, shortness of breath, or difficulty breathing

**OR**

Severe respiratory illness with at least one of the following:

- Clinical or radiographic evidence of pneumonia, **OR**
- Acute respiratory distress syndrome (ARDS).

**AND**

No alternative more likely diagnosis

1. **Laboratory Criteria**

Laboratory evidence using a method approved or authorized by the U.S. Food and Drug Administration (FDA) or designated authority:

*Confirmatory laboratory evidence:*

- Detection of severe acute respiratory syndrome coronavirus 2 ribonucleic acid (SARS-CoV-2 RNA) in a clinical specimen using a molecular amplification detection test

*Presumptive laboratory evidence:*

- Detection of specific antigen in a clinical specimen
- Detection of specific antibody in serum, plasma, or whole blood indicative of a new or recent infection*

**Serologic methods for diagnosis are currently being defined.*

1. **Epidemiologic Linkage**

One or more of the following exposures in the 14 days before onset of symptoms:

- Close contact** with a confirmed or probable case of COVID-19 disease; **OR**
- Close contact** with a person with:
  - clinically compatible illness **AND**
  - linkage to a confirmed case of COVID-19 disease.
- Travel to or residence in an area with sustained, ongoing community transmission of SARS-CoV-2.
- Member of a risk cohort as defined by public health authorities during an outbreak.

***Close contact is defined as being within 6 feet for at least a period of 10 minutes to 30 minutes or more depending upon the exposure. In healthcare settings, this may be defined as exposures of greater than a few minutes or more. Data are insufficient to precisely define the duration of exposure that constitutes prolonged exposure and thus a close contact.*

1. **Other Criteria**

### Vital Records Criteria

- A death certificate that lists COVID-19 disease or SARS-CoV-2 as a cause of death or a significant condition contributing to death
